# Reg4 and its downstream transcriptional activator CD44ICD in stage II and III colorectal cancer

**DOI:** 10.18632/oncotarget.27896

**Published:** 2021-02-16

**Authors:** Jared A. Sninsky, Kumar S. Bishnupuri, Iván González, Nikolaos A. Trikalinos, Ling Chen, Brian K. Dieckgraefe

**Affiliations:** ^1^Division of Gastroenterology, Washington University School of Medicine, Saint Louis, MO 63110, USA; ^2^Division of Pathology and Immunology, Washington University School of Medicine, Saint Louis, MO 63110, USA; ^3^Division of Oncology, Washington University School of Medicine, Saint Louis, MO 63110, USA; ^4^Division of Biostatistics, Washington University School of Medicine, Saint Louis, MO 63110, USA; ^5^Division of Gastroenterology, University of North Carolina School of Medicine, Chapel Hill, NC 27599, USA

**Keywords:** Reg4, CD44, CD44ICD, colorectal cancer

## Abstract

Reg4 is highly expressed in gastrointestinal malignancies and acts as a mitogenic and pro-invasive factor. Our recent works suggest that Reg4 binds with CD44 and induces its proteolytic cleavage to release intra-cytoplasmic domain of CD44 (CD44ICD). The goal of this study is to demonstrate clinical significance of the Reg4-CD44/CD44ICD pathway in stage II/III colon cancer and its association with clinical parameters of aggression. We constructed a tissue microarray (TMA) of 93 stage II/III matched colon adenocarcinoma patients, 23 with recurrent disease. The TMA was immunohistochemically stained for Reg4, CD44, and CD44ICD proteins and analyzed to identify associations with tumor characteristics, recurrence and overall survival. The TMA data analysis showed a significant correlation between Reg4 and CD44 (r^2^ = 0.23, *P* = 0.028), CD44 and CD44ICD (r^2^ = 0.36, *p* = 0.0004), and Reg4 and CD44ICD (r^2^ = 0.45, *p* ≤ 0.0001). Reg4 expression was associated with larger tumor size (r^2^ = 0.23, *p* = 0.026). Although, no association was observed between Reg4, CD44, or CD44ICD expression and disease recurrence, Reg4-positive patients had a median survival of 4 years vs. 7 years for Reg4-negative patients (*p* = 0.04) in patients who recurred. Inhibition of the Reg4-CD44/CD44ICD pathway may be a future therapeutic target for colon cancer patients.

## INTRODUCTION

Colorectal cancer (CRC) is the third most common cancer diagnosed in both men and women in the United States. The American Cancer Society estimates 101,420 new cases of colon cancer, 44,180 new cases of rectal cancer and about 51,020 deaths from CRC in the United States for 2019. While stage I CRC is overwhelmingly curable with resection, metastatic or stage IV disease is commonly fatal with a 5-year survival rate of 15% and requires a multidisciplinary approach with a chemotherapy backbone [1]. For locally advanced (stage II) and node positive (III) disease the approach is still curative, but 5-year survival rates range from 50–80%, with mortality ascribed to recurrent disease [2–4]. After surgical resection, stage II patients are either observed or offered chemotherapy if deemed clinically high-risk, while stage III patients receive anywhere from 3 to 6 months of adjuvant 5-FU based treatment [5]. Chemotherapy in stage II and III disease is hypothesized to treat micro-metastases that account for disease recurrence following surgery [6–8].

Reg4 protein, encoded by a member of the regenerating (Reg) multigene family is a secretory islet-derived protein involved in growth and differentiation of cells, originally discovered through a high throughput assay of an ulcerative colitis library [9]. Reg4 has been demonstrated to precipitate an aggressive neoplastic phenotype marked by increased mortality in CRC patients [10, 11]. Reg4 is associated with anti-apoptotic mechanisms as demonstrated by *in vitro* resistance to 5-FU chemotherapy and radiation [12–14]. While the mechanism of Reg4 has long remained a mystery, our research group has recently observed that exogenous Reg4 acts through the extracellular CD44 receptor and can trigger intramembranous proteolysis of CD44 to release its intracytoplasmic domain (CD44ICD) into the cytoplasm. CD44 then undergoes translocation to the nucleus activating transcription of a series of pro-proliferative, pro-metastatic and anti-apoptotic genes such as KLF4 and SOX-2 (data unpublished). Various studies corroborate that CD44ICD triggers a series of “stemness factors” in the nucleus such as Nanog, Sox-2, and Oct-4 that lead to a pro-metastatic cancer phenotype with increased tumorigenicity [15, 16]. Given the interplay between Reg4 and colorectal cancer aggressiveness, we sought to investigate if expression of Reg4 and its downstream proteins are associated with clinical recurrence and overall survival in stage II and III colorectal cancer patients treated with curative intent.

## RESULTS

### Patient characteristics

Patient characteristics were as follows: Rectal cancer patients were excluded. Age ranged between 50 to 80, without history of neo-adjuvant chemotherapy or radiation prior to resection. Forty-four of these patients were TNM stage 2A, four were 3A, forty were 3B, and five were stage 3C. Of stage II patients, 32% of patients received chemotherapy (14/44) while 100% of stage III CRC patients (49/49) received chemotherapy, consistent with current guidelines. Out of 93 patients, 24 patients had recurrent disease and the other 69 survived at least 5 years after surgical resection without recurrence.

### TMA evidence of Reg4-CD44-CD44ICD pathway

Prior to our principal analysis, we confirmed the homogeneity of protein expression between the two cores. A chi-squared analysis revealed no significant association of percent protein expression or staining intensity between duplicate tumor cores for either Reg4, CD44, or CD44ICD for each patient.

**Figure 1:**
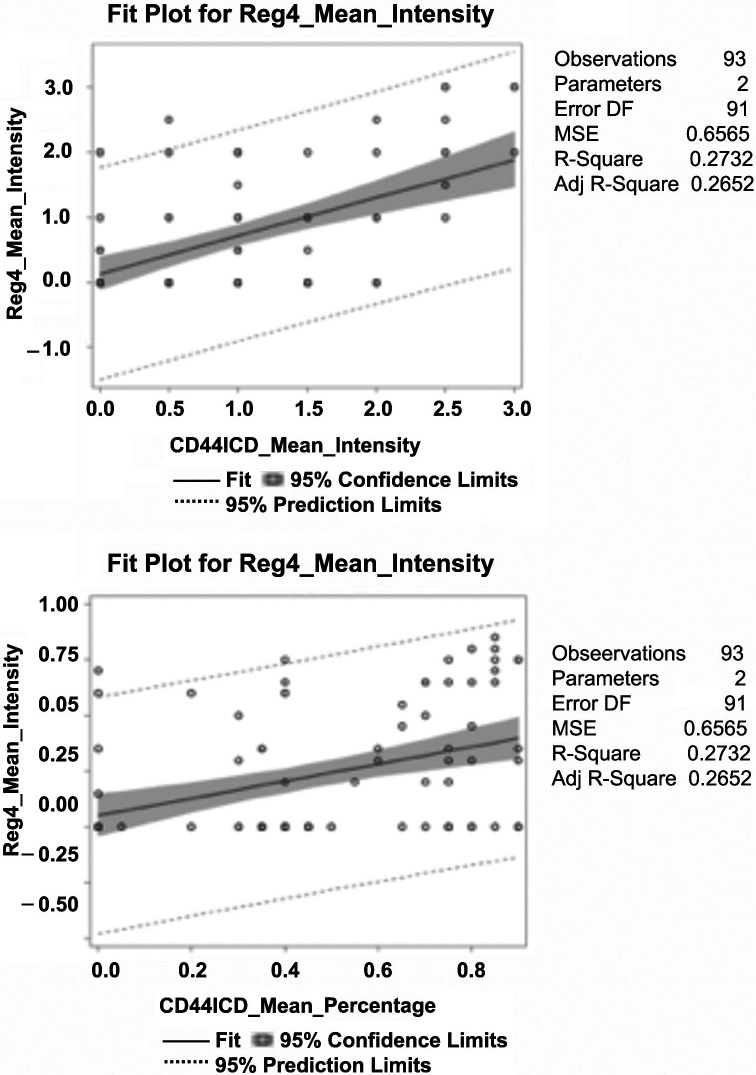
Spearman’s correlation between Reg4, CD44, and CD44ICD protein expression in human CRC patients. Percent mean expression (0–100%) and mean intensity of individual protein expression (0, 1, 2, and 3) was scored blindly by the pathologist. Data analysis shows: a significant correlation of percent mean expression and mean protein intensity between Reg4 and CD44, CD44 and CD44ICD, and Reg4 and CD44ICD. The correlation between Reg4 and CD44ICD was strongest among them.

We then examined the correlation between Reg4, CD44, and CD44ICD for each patient to explore whether this *in vitro* studied mechanism was clinically valid. We utilized a Spearman’s correlation to study the association between each of these proteins using percent mean expression (0–100%), mean intensity (0, 1, 2, 3), and H-score as determined blindly by the pathologist. As shown in Figure 1, a significant Spearman correlation of mean percent expression was observed between Reg4 and CD44 (ρ = 0.23, *P* = 0.025), CD44 and CD44ICD (ρ = 0.31, *p* = 0.0026), and Reg4 and CD44ICD (ρ = 0.42, *p* ≤ .0001). Mean protein intensity also revealed a significant association between Reg4 and CD44 (ρ = 0.23, *P* = 0.028), CD44 and CD44ICD (ρ = 0.36, *p* = 0.0004), and Reg4 and CD44ICD (ρ = 0.45, *p* ≤ .0001). The strongest association in the pathway was observed between Reg4 and CD44ICD.

**Figure 2:**
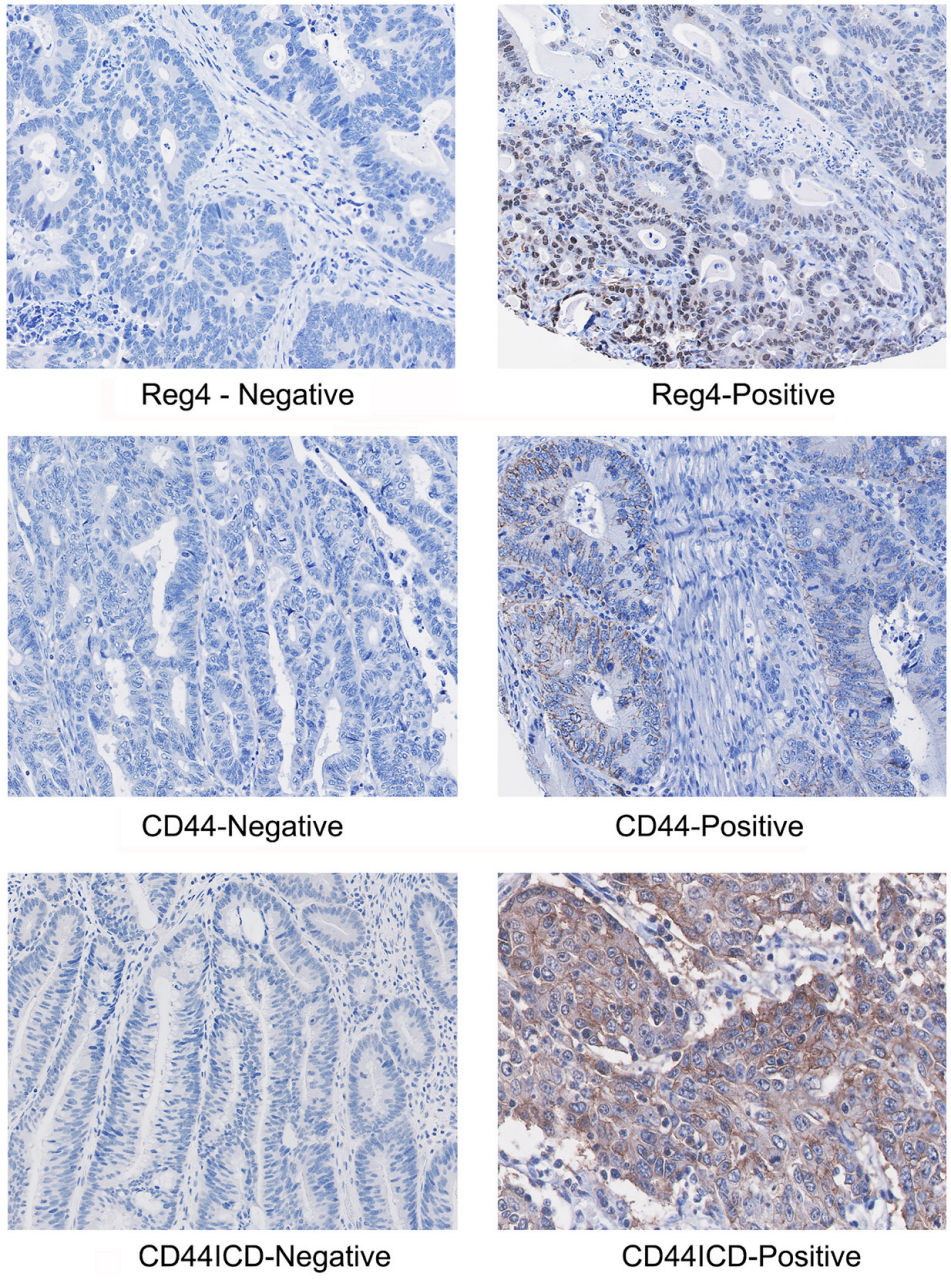
A representative immunostaining expression of Reg4, CD44 and CD44ICD proteins in CRC TMA core showing representative positive and negative samples. Microphotographs shows increased staining of CD44 in membranous part of the colonic cells, whereas increased staining of Reg4 and CD44ICD are localized either in cytoplasmic or/and nuclear parts of the cells.

A representative immunostaining expression of Reg4, CD44 and CD44ICD proteins in our TMA core from representative positive and negative samples is shown in Figure 2. In CRC patients, increased expression of CD44 was localized in the membranous part of the colonic cells, whereas Reg4 and CD44ICD were expressed at higher levels in either cytoplasmic and/or nuclear parts of the cells. This correlated expression of Reg4, CD44 and CD44ICD establishes an important axis of Reg4-CD44-CD44ICD, a hallmark suggesting a crucial role in colorectal cancer biology.

### Protein expression and tumor characteristics

We then looked at accepted prognostic factors and protein expression. Laterality (left vs right) and MMR status had no correlation with expression of the three proteins. MMR deficient patients had an average Reg4 mean expression of 20.5% compared to 34.6% expression in MMR intact patients (*p* = 0.10). However, tumor differentiation did show an association. CD44 mean percent expression (r^2^ = 0.26, *p* = 0.013) and mean intensity (r^2^ = 0.26, *p* = 0.012) both correlated with higher grade tumors. Reg4 mean expression (r^2^ = 0.23, *p* = 0.026) and mean intensity (r^2^ = 0.21, *p* = 0.039) were both associated with larger tumor size. Tumors cores with > 50% Reg4 staining were associated with larger tumors than tumors with < 50% Reg4 staining; 4.46 cm (3.99–4.93) vs. 6.07 cm (4.67–7.47); *p* = 0.043.

### Protein expression and mortality in recurrent disease

**Figure 3:**
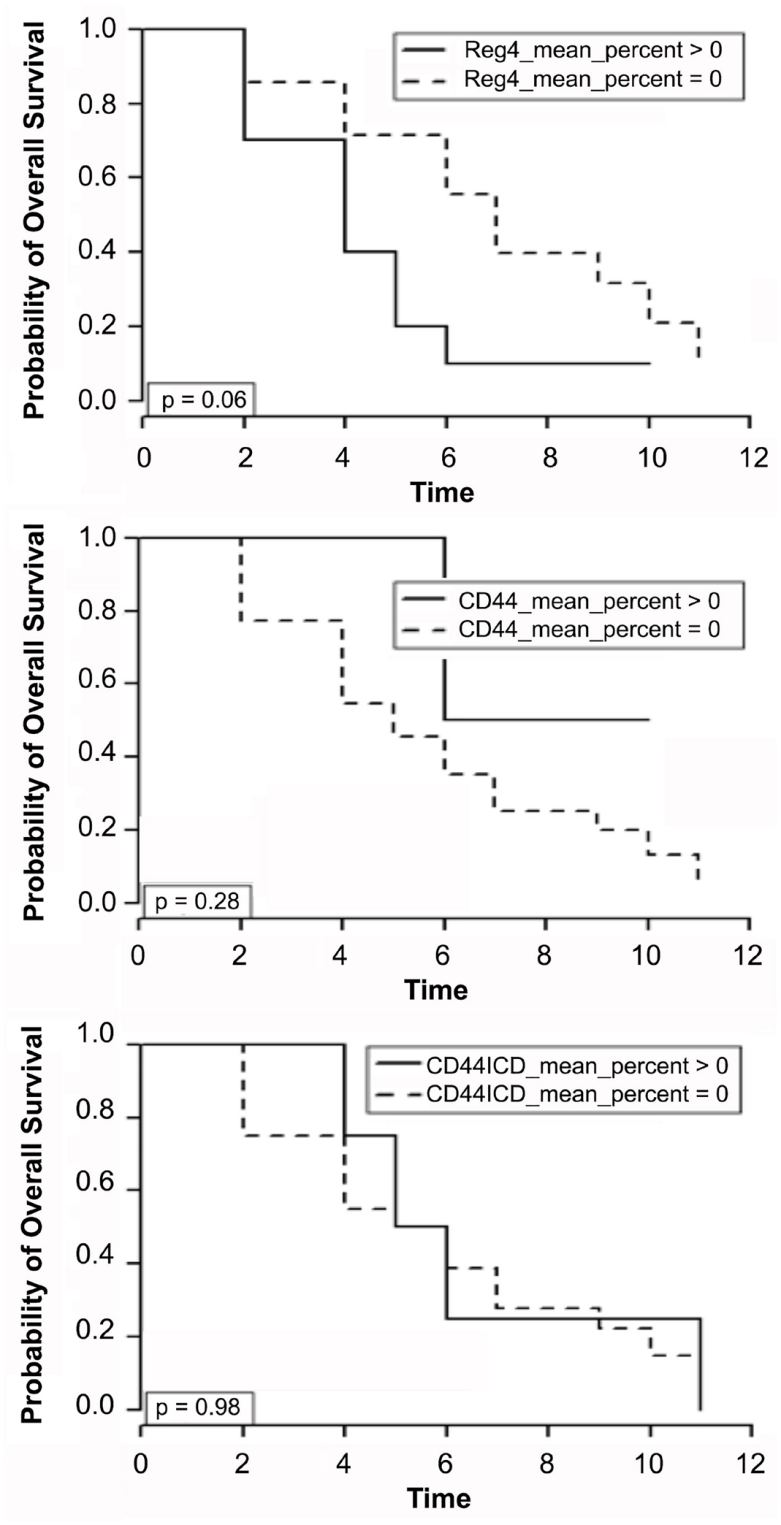
Kaplan-Meier survival curves for patients that had recurrent disease after initial resection (*n* = 24). Protein expression of Reg4, CD44 and CD44ICD from tumor core at initial resection was correlated with time, in years, from initial surgical resection to death. Conditional logistic analysis revealed association of Reg4 with significantly higher mortality in stage II and III patients who developed recurrent disease.

Kaplan-Meier survival curves were constructed for patients that had recurrent disease after initial resection (*n* = 24). Protein expression from tumor core at initial resection was correlated with time, in years, from initial surgical resection to death. No association was found with either CD44 or CD44ICD expression and mortality once patients developed recurrent disease. Conditional logistic analysis revealed no significant association between disease recurrence and mean percent Reg4 (*p* = 0.95), CD44 (*p* = 0.36), or CD44ICD (*p* = 0.58) expression. Furthermore, no significant association with recurrence was detected with mean intensity of Reg4 (*p* = 0.72), CD44 (*p* = 0.30), and CD44ICD (*p* = 0.63) or H-score. However, Reg4 positivity was associated with significantly higher mortality in stage II and III patients who developed recurrent disease. In patients who recurred, Reg4-positive cases at initial resection had a median survival time of 4 years [95% CI: 4–10] versus 7 years [95% CI: 2–5] for Reg4-negative cases (*p* = 0.04). Figure 3 displays the Kaplan-Meier curves relating protein expression with time to death in years after initial surgical resection.

### Peptides inferring CD44ICD activity inhibit CRC cell proliferation and stemness

**Figure 4:**
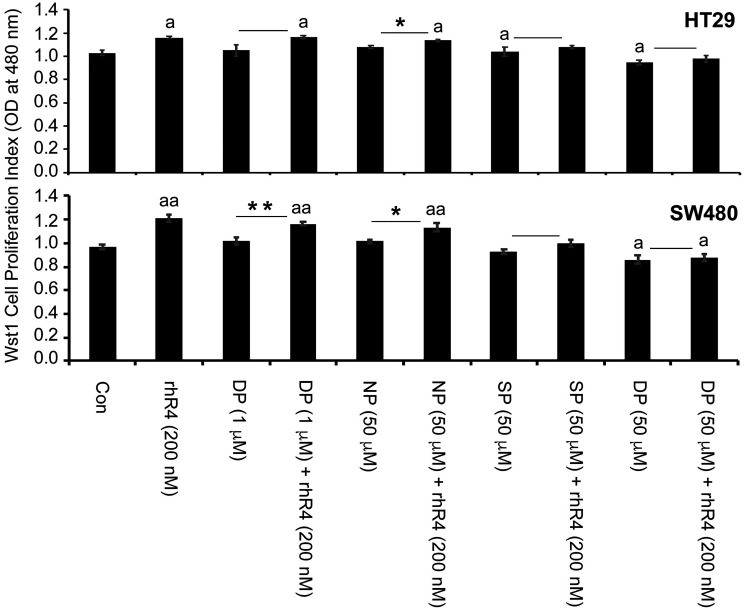
Pen-CD44ICD peptides interfering endogenous nuclear CD44ICD activity inhibit CRC cell proliferation. As assessed by WST-1 cell proliferation assay using HT29 and SW480 CRC cells, treatment of Reg4 increased rate of cell proliferation, whereas Pen-CD44ICD peptides either reduced the rate of cell proliferation and/or blocked the pro-proliferative effect of Reg4. A lower concentration of DP (1 μg/ml) and higher concentration of NP (50 μg/ml) were used as experimental controls showing either no effect on the rate of cell proliferation or no blocking of Reg4-mediated increase in cell proliferation. However, SP (50 μg/ml) significantly blocked Reg4-mediated increase in cell proliferation, and DP (50 μg/ml) significantly reduced the rate of cell proliferation as well as blocked Reg4-mediated increase in cell proliferation. (^a^ *p* < 0.05 vs Con; ^*^ *p* < 0.05; ^**^ *p* < 0.01).

We performed *in vitro* experiments using HT29 and SW480 colorectal cancer cell lines using these peptides to interfere with endogenous CD44ICD and to block Reg4-mediated effects. Following WST1 cell proliferation assay as shown in Figure 4, we observed a Reg4-mediated increase in cell proliferation (^a^ *p* < 0.05). Addition of CD44ICD interfering peptides to the cultures of these cells either reduced the rate of cell proliferation and/or blocked the pro-proliferative effect of Reg4. We used either lower concentration of double phosphorylated Pen-CD44ICD (DP: 1 μg/ml) or higher concentration of non-phosphorylated Pen-CD44ICD (NP: 50 μg/ml) as experimental controls showing either no effect on the rate of cell proliferation or no blocking of Reg4-mediated increase in cell proliferation. However, single phosphorylated Pen-CD44ICD (SP: 50 μg/ml) significantly blocked Reg4-mediated increase in cell proliferation, while double phosphorylated Pen-CD44ICD (DP: 50 mg/ml) significantly reduced the rate of cell proliferation as well as blocked Reg4-mediated effects.

**Figure 5:**
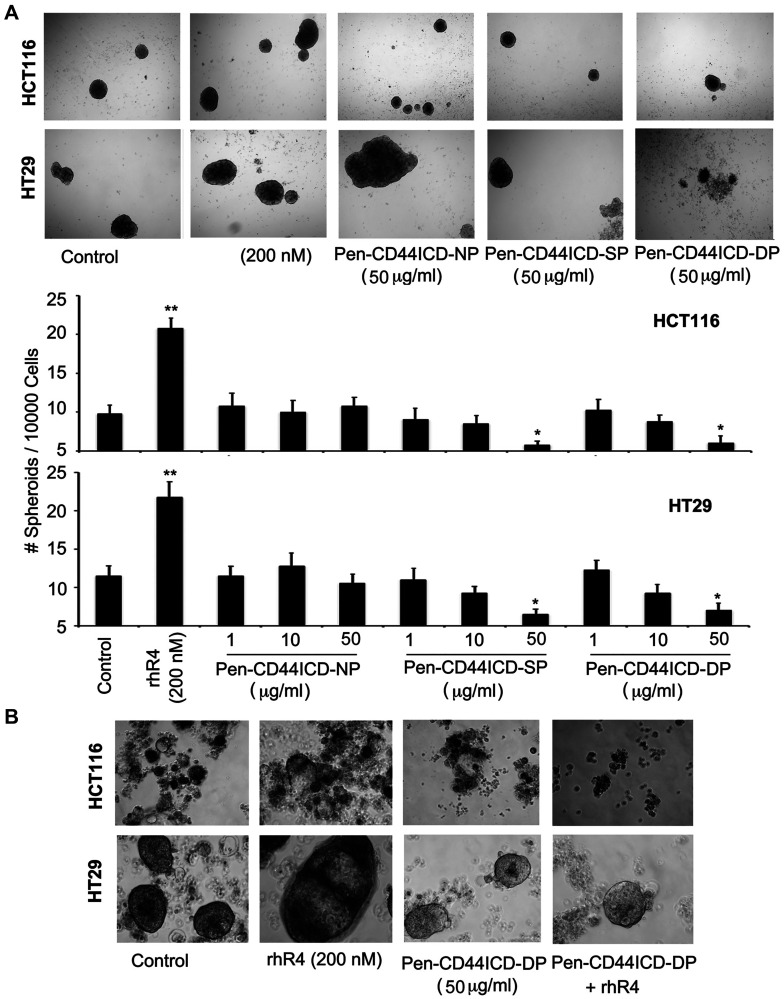
Pen-CD44ICD peptides interfering with nuclear CD44ICD activity inhibit stemness of CRC cells. As assessed by spheroid formation assays using HCT116 and HT29 CRC cells, Reg4 treatment increased the number and size of spheroids whereas higher doses of SP (50 μg/ml) and DP (50 μg/ml) significantly decreased it; no change was observed with an equivalent dose of NP (50 μg/ml). (**A**) Graphical representation of data is shown in lower panel A, where lower doses of NP, SP and DP (1 μg/ml and 10 μg/ml) exhibited no significant changes, hence served as experimental controls. Spheroid cultures of HCT116 and HT29 shown in (**B**) Reg4 further confirmed Reg4-mediated increase in the number and size of spheroids, whereas DP (50 μg/ml) significantly blocked Reg4-mediated effects. (^*^ *p* < 0.05).

We further determined the effect of CD44ICD-interfering peptides in Reg4-mediated survival of CRC stem cells. As assessed by spheroid formation assays using HCT116 and HT29 CRC cells shown in Figure 5, treatments of with a single dose of single phosphorylated Pen-CD44ICD (SP: 50 μg/ml), and double phosphorylated Pen-CD44ICD (DP: 50 μg/ml) led to a significant reduction in the number and size of spheroids, whereas no change was noted with similar treatments of non-phosphorylated Pen-CD44ICD (NP) (panel A: upper). A graphical representation of data is shown in lower panel A, where lower dose of NP, SP and DP (1 μg/ml and 10 μg/ml) showing no significant changes in the number and size of spheroids served as additional experimental controls. As an extension of this experiment, we used double phosphorylated Pen-CD44ICD (DP: 50 μg/ml) with and without Reg4 and performed similar spheroid formation assays using HCT116 and HT29 CRC cells. Assessing the number and size of spheroids in culture, Pen-CD44ICD (DP) treatment blocked Reg4-mediated effect (panel B).

Results of this study demonstrated an involvement of Reg4-mediated generation of nuclear CD44ICD regulating proliferation and stemness of human CRC cells. These results also identify CD44ICD as a potential target of therapeutic interventions for human CRC patients.

## DISCUSSION

In this study, we constructed a TMA of 94 stage II and III colon cancer patients matched by patient/tumor characteristics and disease recurrence. We immunohistochemically stained our TMA with three proteins integral to the Reg4 pathway: Reg4, CD44, and CD44ICD and looked into associations with clinical parameters of tumor aggression. We found that Reg4 expression is associated with larger tumors and higher mortality in metastatic disease, but not with recurrence risk per se. We also confirmed that the Reg4-CD44-CD44ICD is a relevant pathway in the clinical setting.

Reg4 is a secretory islet-derived protein involved in growth and differentiation originally discovered through a high throughput assay of an ulcerative colitis library [9, 17]. Reg4 exerts its effects in a paracrine and autocrine fashion precipitating an aggressive neoplastic phenotype marked by increased mortality in CRC patients, as confirmed in our study [18, 19]. *In vitro* studies have shown that Reg4 promotes migration and invasion of CRC tumor cells with reversal of this phenomenon upon treatment with anti-Reg4 antibody [18, 20]. Mouse models have demonstrated Reg4 to promote gastric cancer adhesion to murine peritoneum *in vivo* [21] and a patient cohort associated Reg4 levels of peritoneal washings with peritoneal micro-metastasis [22]. In our lab, we successfully used a CD44ICD interrupting peptide to inhibit Reg4 induced proliferation. Reg4 acts as a growth factor with exogenous Reg4 stimulating growth and *in vitro* invasiveness of colon cancer cells [23–25].

The role of Reg4 and treatment resistance is still being elucidated. Our lab has previously demonstrated that exogenous Reg4 added to human colon adenocarcinoma cells leads to upregulation of the anti-apoptotic gene bcl-2 and induces resistance *in vitro* to ionizing irradiation [13] and 5-FU chemotherapy [12]. In 2002, a study by Violette et al. demonstrated that the Reg4 gene is upregulated in drug-resistant HT-29 colon cancer cell lines and downregulated in drug sensitive cell lines [12]. In gastric cancer lines, SiRNA knockdown of Reg4 promoted 5-FU induced apoptosis with no change in 5-FU metabolites inferring a novel resistance pathway [26]. Recently, knockout of Reg4 in mice suppressed colon cancer stem cell markers and in a patient cohort, Reg4 was significantly associated with CRC stem cell markers [27]. These studies suggest that high levels of Reg4 in colon adenocarcinoma confer a survival advantage when exposed to 5-FU. Our study also showed that in stage II and III patients who developed recurrent disease, Reg4 expression was associated with significantly higher mortality.

Although we didn’t observe an association between Reg4 expression and recurrence risk in this study, possibly due to limited number of samples, our results clearly demonstrated that the expression of Reg4 promotes tumor growth and chemoresistance. In 2015, Zhu et al. demonstrated that Reg4 expression in combination with MMP-7 in clinical CRC resections was significantly associated with lymph node disease, T stage, clinical stage, metastasis and decreased survival [10]. However, this study looked into a much broader population, including very few (< 10%) metastatic patients and did not perform a subgroup analysis to determine what drove the increased mortality [10]. A cohort of 202 CRC patients demonstrated that Reg4 mRNA expression in tumors was an independent predictor of survival at 5 years [11]. Interestingly, in prostate cancer, Reg4 has been shown to be an independent predictor of relapse after prostatectomy [28] and in gastric cancer associated significantly with tumor invasion depth, lymph node metastasis, and increased mortality [29]. In gastric cancer, *in vitro* Reg4 antibody has inhibited gastric cancer proliferation and enhanced the apoptotic effect of 5-FU [30].

We propose that Reg4 promotes local invasion, proliferation, and properties of chemo-resistance; but is not directly implicated in the development of distant metastasis. This can explain why Reg4 positive patients tend to have less differentiated and larger tumors yet are not more likely to recur after resection in early-stage disease. In the metastatic setting, tumors that express high levels of Reg4 are less responsive to 5-FU regimens, leading to increased mortality. The idea of stage specific prognostic biomarkers in cancer is not new [31]. For example, Kirsten rat sarcoma virus (KRAS) mutations are associated with worse disease-free survival [HR 1.29 *p* = .008] amongst stage III CRC patients, but no survival difference in stage II disease. KRAS mutations are used to choose treatments in the metastatic and inoperable setting [32–34]. Microsatellite instability is associated with a better prognosis in stage II and III colon cancer; but has no prognostic implications in metastatic disease treated with standard therapy [35]; although this is currently changing with the use of immunotherapy [36, 37]. In other malignancies such as melanoma, BRAF positivity does not factor into treatment options for node negative disease and has no known prognostic significance, but it informs management in the metastatic setting [38, 39].

In this study, we also found a significant association between Reg4, CD44, and CD44ICD; demonstrating for the first time that this pathway is biologically relevant outside of cancer cell lines alone. While the exact mechanism of Reg4 has long remained a mystery, our lab (unpublished data) has noted that exogenous Reg4 acts through the extracellular CD44 receptor. This interaction is hypothesized to trigger intramembranous proteolysis of CD44 and release of CD44ICD, or CD44 intracytoplasmic domain, to the nucleus activating a series of pro-metastatic and anti-apoptotic genes. Recent *in vitro* data from our lab reveals a direct interaction between Reg4 and the CD44, using a biotinylated Reg4 pull down assay and mass spectrometry. Our work has further demonstrated that Reg4 induces proteolytic cleavage of CD44, releasing CD44ICD into the nucleus. The direct interaction between these proteins works through intramembrane proteolysis (RIP)-based signaling, recently recognized as a common signaling mechanism used by a number of type 1 transmembrane proteins including Notch, Delta and CD44 [40]. Analogous to Notch signaling, y-secretase releases a 74aa CD44 intracellular domain fragment (CD44ICD) into the nucleus [41, 42].

The first description of phorbol ester-induced proteolytic release of CD44ICD was reported in glioblastoma cells in 2001 [43]. Other neoplasms such as papillary thyroid carcinomas have been found to express CD44ICD to sustain cell proliferation via CREB-dependent transcriptional activation of Cyclin D1. Gonzalez et al. (2012) characterizes the CD44- CD44ICD pathway and its downstream genes, defining its critical role in various glycolytic pathways and cancer stem cell knockdown of CD44 by RNA interference or y-secretase inhibitors (GSIs) blocked proliferation, which was restored by CD44ICD overexpression. CD44ICD contains a nuclear localization signal and is a potent activator of oncogenic transcription [16, 44, 45]. Studies suggest that CD44ICD triggers a series of “stemness factors” in the nucleus such as nanog, sox-2, and oct-4 that lead to a pro-metastatic cancer phenotype with increased tumorigenicity [46]. Inhibition of CD44 proteolytic cleavage blocks the pro-proliferative properties of Reg4 in the lab, demonstrating that the CD44-CD44ICD pathway is the driving mechanism of Reg4 tumorigenicity. The significant association found in our study between Reg4, CD44, and CD44ICD gives biological credibility to this pathway within a clinical cohort of colon adenocarcinoma patients. This dose dependent relationship was seen both in percent field of protein expression and mean intensity of protein expression, suggesting the direct interaction of these proteins in a pathway rather than a mere association.

Our study was limited by non-randomization and the size of the cohort with 94 total matched patients, 24 of which had recurrent disease. Due to the retrospective nature, our data was incomplete and subject to sampling bias. Many of our patients had pathology analysis before BRAF and KRAS testing was standardized, and thus any association between survival, the Reg4 pathway and these proteins could not be assessed. The recurrence data from the TMA was reported by distant, not local metastasis and we did not have disease-specific mortality. However, our demographics including sex, recurrence rates, survivals, and microsatellite instability percentages did mirror that of the general population, leading us to believe that our TMA could serve as a representative sample. More prospective studies in an unselected population will be needed to confirm these findings.

In conclusion, we have shown that in colon adenocarcinomas that express high levels of Reg4, this pathway becomes a major determinant of resistance and can affect survival in the metastatic setting. Our TMA analysis has also clinically validated the presence and significance of the Reg4-CD44-CD44ICD signaling pathway in tumor cells of human CRC patients. Our *in vitro* data shows that Reg4-CD44ICD signaling regulates proliferation and survival of the cancer stem cells, hence providing a unifying explanation for poor clinical outcomes with Reg4 and CD44/CD44ICD. We plan to validate these findings within a larger cohort of patients and continue to expand our work by interfering with this pathway to improve treatment in metastatic disease.

## MATERIALS AND METHODS

### Patients for tissue microarray

A total of 93 stage II and III patients with colon adenocarcinoma who underwent surgical resection at Siteman Cancer Center between 2005 and 2012 were chosen to construct tissue microarray (TMA) slides of 4 microns sections. Each TMA included 6 control tissue cores including: tonsil, appendix, kidney, nipple, brain, and placenta. Reg4 expression is upregulated in the appendix and thus served as the specificity control.

The sources of these tissue cores were IRB-approved closed human studies (IRB #201802060, Wash U St. Louis), informed consent previously obtained from patients to enter registry. The formalin-fixed and paraffin-embedded (FFPE) tissue blocks were available for all cases. All the cases were re-reviewed by a pathologist and two separate 1mm tumor cores were obtained from distinct sections to account for tumor heterogeneity. These TMAs were constructed at the core facilities of Washington University School of Public Health under the auspices of Thomas Walsh.

### Study design

This was a retrospective translational cohort study using tissue specimens from patients that have undergone surgical resection at the Siteman Cancer center from 2005–2012, with paired demographic information and clinical outcome data. Patients were selected from the Siteman Cancer registry database chronologically starting in 2005, where the first three patients to meet matching criteria were included after a recurrent case was identified. A sample size of 94 patients was derived assuming 50% positive CD44ICD samples, with a power ≥ 0.80 to detect a hazard ratio of 2.5, assuming a 3-year DFS 72% in patients with colon cancer stage III. The power analysis was based on two-sample log-rank test at a significance level 0.05.

Each recurrent case was paired with 1–4 non-recurrent cases matched by TNM stage, age (+/– 5 years), sex, and chemotherapy administration (yes/no). Patients in the database are followed annually for life, with recurrence and vital status confirmed through the EMR by the Siteman Cancer registry if followed at Washington University or through letter/phone contact if at an outside clinical practice. The clinical endpoint examined was recurrent disease as defined by the primary oncologist based on imaging studies.

The primary variable was CD44ICD, CD44, and Reg4 immunohistochemical staining determined by a pathologist, scored for intensity and percent staining. Additionally, an H-score or “histology” score was calculated for each protein, derived by multiplying percent staining of a protein by its intensity score. CD44ICD nuclear staining was chosen as it most closely reflected our cell-line model of CD44ICD activating “stemness” genes in the nucleus. The matched variables were age, sex, chemotherapy, and TNM stage. Specific chemotherapy regimen (e.g., FOLFOX or CapeOx) was not matched, but all patients received some form of 5-FU based chemotherapy. We also recorded multiple co-variables such as tumor size, MMR status, tumor laterality (left or right sided), tumor histology (poor, moderate, well), and years to recurrence. MMR status was reviewed to determine if there was any relationship between the Reg4 and microsatellite instability pathways. MSI-H status has also been associated with distinct recurrence rates by location and worse survival after recurrence, so this variable was identified to avoid a potential confounder [47]. The overall demographics of patients included in this TMA are shown in Table 1.

**Table 1:** TMA demographics

**MMR Status**	
MMR intact	80 (86%)
MMR deficient	13 (14%)
**Location**	
Right	49 (53%)
Left	44 (47%)
**Differentiation**	
Poor	19 (21%)
Moderate	67 (73%)
Well	6 (7%)
**TMN Stage**	
2A	44 (47%)
3A	4 (4.3%)
3B	40 (43%)
3C	5 (5%)
**Size**	Average 4.8 cm [2.35–7.25]
**Age**	Average 65 y [55.4–74.6]
**Sex**	
Female	46 (49%)
Male	47 (51%)
**Chemotherapy**	
No	30 (32%)
Yes	63 (68%)
**Recurrence**	
No	69 (74%)
Yes	24 (26%)

The histology and tumor size were identified based on the official pathology report in the medical record. MMR status was identified through IHC staining of our TMA for MLH1/PMS2 and MSH2/MSH6 since most tumor sections were resected prior to standardized testing for microsatellite instability. Studies have corroborated that MMR status can be detected accurately by TMA in colon cancer [48, 49].

### Antibodies

A rabbit monoclonal antibody specific to panCD44 was purchased from Cell Signaling Technology (Danvers, MA, USA). Affinity-purified polyclonal antibodies from New Zealand rabbit specific to human Reg4 peptide (sequence: CRSWSGKSMGGNKH; immunogen: peptide-KLH conjugate) and from goat specific to human CD44ICD peptide (sequence: AVEDRKPSGLNGEAC; immunogen: Peptide-KLH conjugate) were custom synthesized and standardized at GeneScript (Piscataway, NJ, USA). These antibodies were used for immunohistochemical staining of TMAs.

### Penetratin-CD44ICD peptide

**Figure 6:**
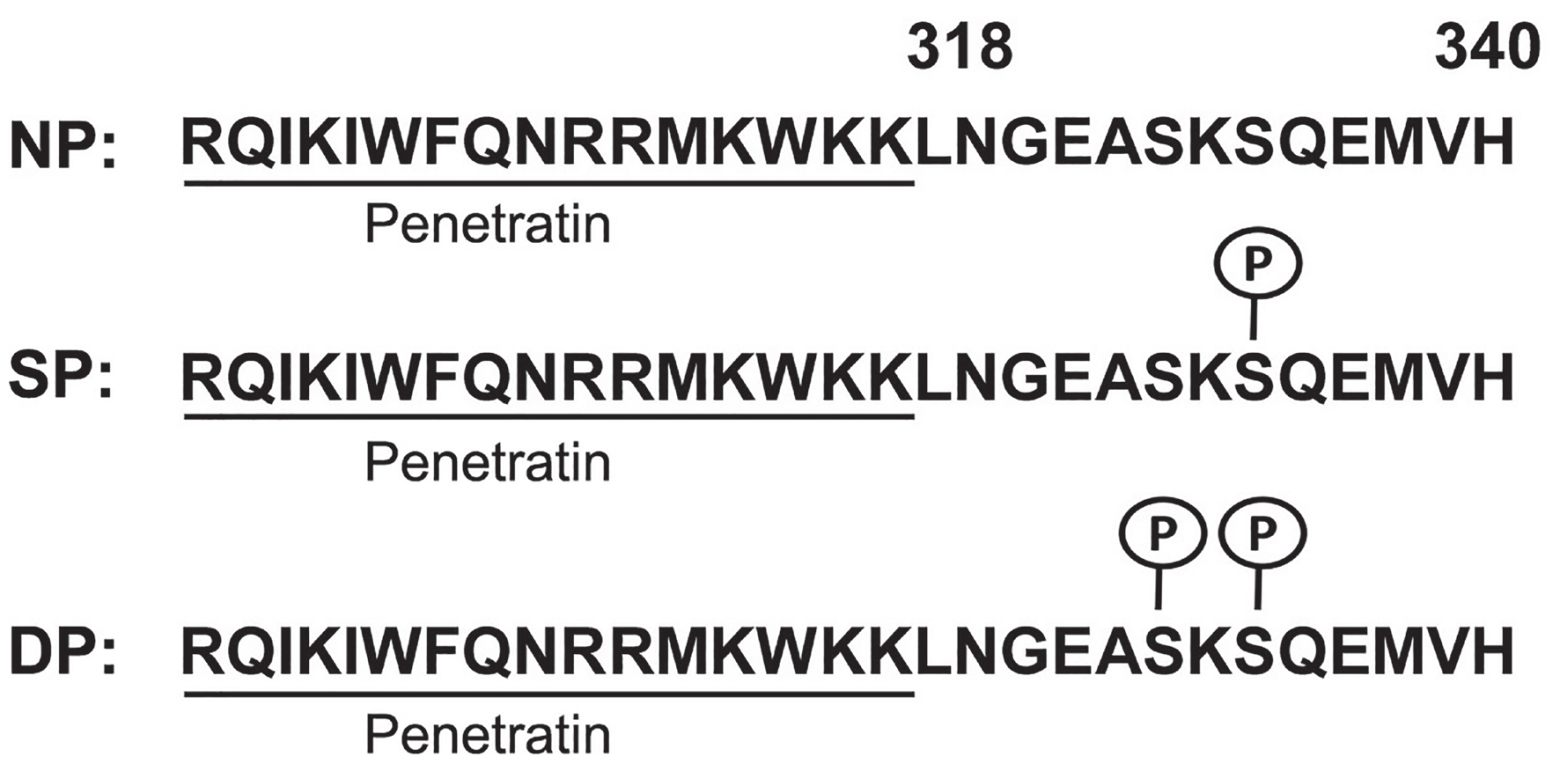
Generation of Penetratin-CD44ICD peptides. All peptides comprise 16 amino acid Penetrating sequence (underlined) followed by amino acid 318–340 from the CD44 intra-cytoplasmic domain (CD44ICD). Three different constructs of Pen-CD44ICD peptides were synthesized: non-phosphorylated (NP), with single phosphoserine at position 325 (SP) and with double phosphoserine at positions 323 and 325 (DP).

We chose the most conserved amino acid sequence of CD44ICD mature peptide (conserved through primitive species to human), and custom synthesized three variants of CD44ICD peptides with an attachment of short 16 amino acid sequence of Penetratin [RQIKIWFQNRRMKWKK] to facilitate their entry into the nucleus of C44-positive cells to interfere with the activity of host CD44ICD (Invitrogen, Carlsbad, CA, USA). The carboxyterminus residues were amidated and a proportion of each peptide was synthesized with a ‘Biotin’ group at the amino-terminus. Peptides were either non-phosphorylated (NP) or were synthesized with single phosphoserine at position 325 (SP), or with double phosphoserine at positions 323 and 325 (DP) as shown in Figure 6.

### Immunohistochemistry

Endogenous peroxide in deparaffinized TMA slides were quenched in 1% Hydrogen Peroxide/Methanol and then subjected to antigen retrieval by steaming in Diva Decloaking Solution (Biocare Medical, Walnut Creek, CA, USA) in a pressure cooker at 15PSI for 3 minutes. Slides were then blocked with Avidin/Biotin blocking solution (Biocare) and incubated with primary antibodies overnight (CD44ICD 1:500, CD44 1:400, Reg IV 1:500). The next day, slides were incubated in respective secondary antibodies (1:1000) followed by incubation in SA-HRP solution (1:1000; Jackson Immunoresearch). Slides were finally stained with Betazid DAB (Biocare), counterstained with hematoxylin, (7211, Richard Allan, Kalamazoo, MI, USA) and mounted to observe/score under phase-contrast microscope.

Prior to our principal analysis, we confirmed the homogeneity of protein expression between the two cores (TMA A and TMA B) for each patient. A chi-squared analysis revealed no significant difference between percent protein expression or staining intensity between the two cores for either Reg4, CD44, or CD44ICD. The TMA was scored by a pathologist blinded to all clinical data, who characterized location of staining (membranous, cytoplasmic, nuclear), intensity of staining (weak, moderate, strong), and percent of field with positive staining. Additionally, nuclear CD44ICD was quantified using the Allred scoring system, an accepted scoring system used in nuclear hormonal quantification in breast cancer that includes intensity and percentage of nuclear staining.

### Cell culture and reagents

Mycoplasma-tested human colorectal cancer (CRC) cell lines (HCT116, SW480 and HT29; American Type Culture Collection, Manassas, VA, USA) were grown in DMEM media containing 10% heat inactivated fetal bovine serum (All from GIBCO by Life Technologies, Grand Island, NY, USA). Cell lines used for experiments were from passage number 3–30 at approximately 70–80% confluency.

### Cell proliferation assay

CRC cell proliferation was assessed by WST-1 assay (Roche, Indianapolis, IN, USA). Briefly, the stable tetrazolium salt WST-1 is cleaved to a soluble formazan in viable cells. Therefore, the amount of formazan dye formed directly correlates to the number of metabolically active cells in the cultures. CRC cells (HT29 and SW480) grown in a 96-well culture plate were incubated with the WST-1 reagent for 4 hours. The formazan dye formed is then quantitated with a Synergy 2 multi-well spectrophotometer (BioTek, Winooski, VT). The measured absorbance directly correlates to the number of viable proliferative cells.

### Spheroid forming assay

The spheroid forming culture is the most widely used *in vitro* functional assay for assessing growth of cancer stem cells [50]. CRC and PC cells were seeded in media containing EGF (1 ng/ml; R&D Systems, Minneapolis, MN, USA), insulin (0.4 μg/ml; Life Technologies, Grand Island, NY, USA) and Rock Inhibitor (10 μM; American Type Culture Collection, Manassas, VA) into the wells of low adhesion culture plate (Corning, NY, USA). Cells were grown 12 days in above-mentioned culture media and the number and size of growing spheroids were quantified microscopically.

### Statistical analysis

Spearman’s correlation was used to study the association between individual proteins using percent mean expression (0–100%) and mean intensity (0, 1, 2, 3) as scored blindly by the pathologist. Survival time was defined as time from initial surgical resection to death. Product-limit survival estimates were obtained to estimate survival probability and log-rank tests were used to compare survival probability between groups. Conditional logistic regression analysis was performed to examine association between disease recurrence and protein expression and intensity. All statistical tests were two-sided at significance level 0.05 and analysis was conducted using SAS9.4 (SAS Inc, Cary, NC, USA).

## Abbreviations

*Reg*: Regenerating
*Reg4*: Regenerating gene 4
Reg4: Protein encoded by *Reg4* gene
CRC: Colorectal cancer
CRT: Chemoradiotherapy
GI: Gastrointestinal
CD44ICD: Intracytoplasmic domain of CD44
